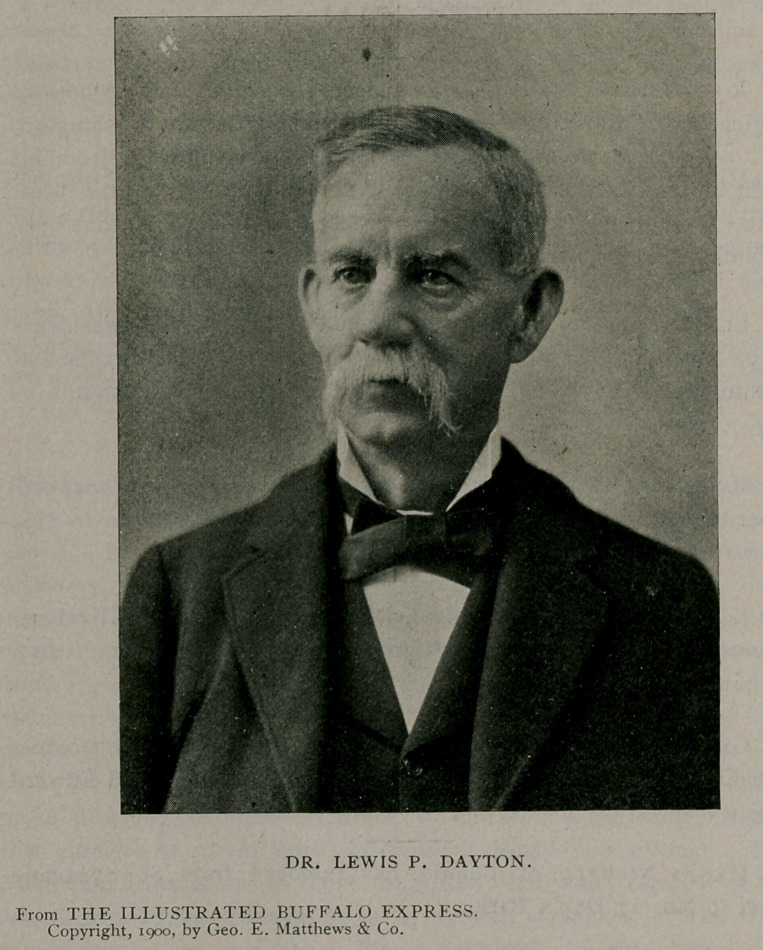# Dr. Lewis P. Dayton

**Published:** 1900-06

**Authors:** 


### OBITUARY.

Dr. Lewis P. Dayton, former Mayor of Buffalo and for many years a leading physician of this city, died at his home on Bryant Street, on May 14, 1900, after an illness of eight years. Born in Eden, Erie

County, in 1819, he was graduated from the Springville Academy in 1840 and from the Medical College at Geneva in 1845, in which year he settled in Buffalo. From that time on he ranked as one of Buffalo's leading physicians. He was identified with many movements begun by the Medical Society of the County of Erie, and was

DR. LEWIS P. DAYTON.

From THE ILLUSTRATED BUFFALO EXPRESS.
Copyright, 1900, by Geo. E. Matthews & Co.

recognised as the dean of the fraternity which existed in Buffalo's early days.

In 1845, Dr. Dayton was elected school commissioner for the town of Black Rock. In 1849 he joined the Medical Society of the County of Erie, was elected vice-president in 1858; and served as president in 1859. During the years 1855, 1856, 1857, 1858, 1862,

1863 and 1864, he represented the old 12th Ward (now the 25th) in the Board of Aldermen. He was Member of Assembly from the Third District in 1865 and 1866, and was Mayor in 1874. He was twice County Clerk and served one term as County Treasurer.

## Figures and Tables

**Figure f1:**